# *N*-Benzoyl-4-chloro­benzene­sulfonamide

**DOI:** 10.1107/S160053681000783X

**Published:** 2010-03-06

**Authors:** P. A. Suchetan, B. Thimme Gowda, Sabine Foro, Hartmut Fuess

**Affiliations:** a Department of Chemistry, Mangalore University, Mangalagangotri 574 199, Mangalore, India; b Institute of Materials Science, Darmstadt University of Technology, Petersenstrasse 23, D-64287 Darmstadt, Germany

## Abstract

The asymmetric unit of the title compound, C_13_H_10_ClNO_3_S, contains two independent mol­ecules. The mol­ecules have C—S—N—C torsion angles of −70.0 (2) and 61.3 (2)° for mol­ecules 1 and 2, respectively. The dihedral angles between the sulfonyl benzene rings and the –SO_2_—NH—C—O segments are 72.0 (1) and 77.3 (1)° for mol­ecules 1 and 2, respectively, and the dihedral angles between the sulfonyl and the benzoyl benzene rings are 62.8 (1) and 78.6 (1)°, respectively. In the crystal, mol­ecules 1 and 2 are linked by pairs of N—H⋯O hydrogen bonds, forming inversion dimers.

## Related literature

For background to our study of the effect of ring and side-chain substituents on the crystal structures of *N*-aromatic sulfonamides and for similar structures, see: Gowda *et al.* (2009 ▶; 2010 ▶); Suchetan *et al.* (2009 ▶).
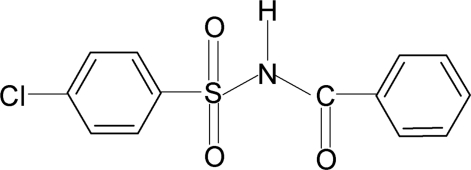


## Experimental

### 

#### Crystal data

C_13_H_10_ClNO_3_S
*M* *_r_* = 295.73
Triclinic, 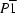

*a* = 9.138 (1) Å
*b* = 12.026 (2) Å
*c* = 12.512 (2) Å
α = 91.15 (1)°
β = 93.53 (1)°
γ = 107.40 (2)°
*V* = 1308.5 (3) Å^3^
*Z* = 4
Cu *K*α radiation
μ = 4.12 mm^−1^
*T* = 299 K
0.50 × 0.40 × 0.40 mm

#### Data collection

Enraf–Nonius CAD-4 diffractometer
Absorption correction: ψ scan North *et al.*, 1968 ▶ *T*_min_ = 0.233, *T*_max_ = 0.290
9165 measured reflections
4655 independent reflections
3966 reflections with *I* > 2σ(*I*)
*R*_int_ = 0.044
3 standard reflections every 120 min  intensity decay: 2.0%

#### Refinement

*R*[*F* ^2^ > 2σ(*F* ^2^)] = 0.044
*wR*(*F* ^2^) = 0.125
*S* = 1.05
4655 reflections
350 parameters
2 restraints
H atoms treated by a mixture of independent and constrained refinement
Δρ_max_ = 0.34 e Å^−3^
Δρ_min_ = −0.34 e Å^−3^

### 

Data collection: *CAD-4-PC* (Enraf–Nonius, 1996 ▶); cell refinement: *CAD-4-PC*; data reduction: *REDU4* (Stoe & Cie, 1987 ▶); program(s) used to solve structure: *SHELXS97* (Sheldrick, 2008 ▶); program(s) used to refine structure: *SHELXL97* (Sheldrick, 2008 ▶); molecular graphics: *PLATON* (Spek, 2009 ▶); software used to prepare material for publication: *SHELXL97*.

## Supplementary Material

Crystal structure: contains datablocks I, global. DOI: 10.1107/S160053681000783X/fk2014sup1.cif

Structure factors: contains datablocks I. DOI: 10.1107/S160053681000783X/fk2014Isup2.hkl

Additional supplementary materials:  crystallographic information; 3D view; checkCIF report

## Figures and Tables

**Table 1:** Hydrogen-bond geometry (Å, °)

*D*—H⋯*A*	*D*—H	H⋯*A*	*D*⋯*A*	*D*—H⋯*A*
N1—H1*N*⋯O1^i^	0.84 (2)	2.16 (2)	2.967 (3)	161 (3)
N2—H2*N*⋯O4^ii^	0.83 (2)	2.15 (2)	2.962 (3)	164 (3)

Symmetry codes: (i) 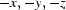; (ii) 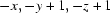.

**Fig. 1.**
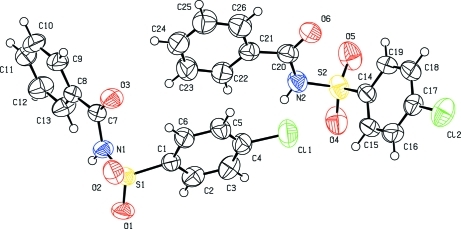
Molecular structure of the title compound, showing the atom-labelling scheme. Displacement ellipsoids are drawn at the 50% probability level.

**Fig. 2.**
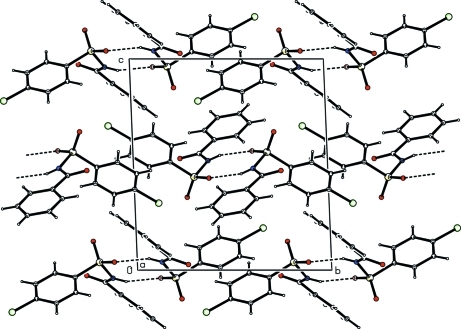
Molecular packing in the title compound. Hydrogen bonds are shown as dashed lines.

## supplementary crystallographic information

### Comment

Diaryl acylsulfonamides are known as potent antitumor agents against a broad spectrum of human tumor xenografts in nude mice. As a part of studying the effect of ring and the side chain substituents on the crystal structures of *N*-aromatic sulfonamides (Gowda *et al.*, 2009; 2010; Suchetan *et al.*, 2009), the structure of *N*-(benzoyl)4-chlorobenzenesulfonamide (I) has been determined. The asymmetric unit of the structure contains two independent molecules (Fig.1). The conformations of the N—H bonds in the C—SO_2_—NH—C(O) segments are *anti* to the C=O bonds, similar to those observed in *N*-(benzoyl)benzenesulfonamide (II) (Gowda *et al.*, 2009), *N*-(benzoyl)2-chlorobenzenesulfonamide (III) (Gowda *et al.*, 2010) and *N*-(4-chlorobenzoyl)benzenesulfonamide (IV)(Suchetan *et al.*, 2009).

The molecules are twisted at the *S* atoms with the torsional angles of -70.0 (2)° and 61.3 (2)° in the two independent molecules. The dihedral angles between the sulfonyl benzene rings and the —SO_2_—NH—C—O segments are 72.0 (1)° (molecule 1) and 77.3 (1)° (molecule 2), compared to the values of 86.5 (1) in (II), 87.3 (1)° in (III) and 75.7 (1)° in (IV). Furthermore, the dihedral angles between the benzene rings are 62.8 (1)° (molecule 1) and 78.6 (1)° (molecule 2), compared to the values of 80.3 (1) in (II), 73.3 (1)° in (III) and 68.6 (1)° in (IV). The packing of molecules linked by N—H···O(S) hydrogen bonds (Table 1) is shown in Fig. 2.

### Experimental

The title compound was prepared by refluxing a mixture of benzoic acid, 4-chlorobenzenesulfonamide and phosphorous oxy chloride for 5 h on a water bath. The resultant mixture was cooled and poured into ice cold water. The solid, *N*-(benzoyl)4-chlorobenzenesulfonamide obtained was filtered, washed thoroughly with water and then dissolved in sodium bicarbonate solution. The compound was later reprecipitated by acidifying the filtered solution with dilute HCl. The filtered and dried compound was recrystallized to the constant melting point.

Prism like colourless single crystals of the title compound used in X-ray diffraction studies were grown from a slow evaporation of its toluene solution at room temperature.

### Refinement

The H atoms of the NH groups were located in a difference map and later restrained to the distance N—H = 0.86 (2) Å. The other H atoms were positioned with idealized geometry using a riding model with C—H = 0.93 Å. All H atoms were refined with isotropic displacement parameters (set to 1.2 times of the U_eq_ of the parent atom).

### Crystal data

**Table:**

C_13_H_10_ClNO_3_S	*Z* = 4
*M**_r_* = 295.73	*F*(000) = 608
Triclinic, *P*1	*D*_x_ = 1.501 Mg m^−^^3^
Hall symbol: -P 1	Cu *K*α radiation, λ = 1.54180 Å
*a* = 9.138 (1) Å	Cell parameters from 25 reflections
*b* = 12.026 (2) Å	θ = 6.3–20.7°
*c* = 12.512 (2) Å	µ = 4.12 mm^−^^1^
α = 91.15 (1)°	*T* = 299 K
β = 93.53 (1)°	Prism, colourless
γ = 107.40 (2)°	0.50 × 0.40 × 0.40 mm
*V* = 1308.5 (3) Å^3^	

### Data collection

**Table:**

Enraf–Nonius CAD-4 diffractometer	3966 reflections with *I* > 2σ(*I*)
Radiation source: fine-focus sealed tube	*R*_int_ = 0.044
graphite	θ_max_ = 67.0°, θ_min_ = 3.5°
ω/2θ scans	*h* = −10→10
Absorption correction: ψ scan North *et al.*, 1968	*k* = −14→14
*T*_min_ = 0.233, *T*_max_ = 0.290	*l* = −14→14
9165 measured reflections	3 standard reflections every 120 min
4655 independent reflections	intensity decay: 2.0%

### Refinement

**Table:**

Refinement on *F*^2^	Secondary atom site location: difference Fourier map
Least-squares matrix: full	Hydrogen site location: inferred from neighbouring sites
*R*[*F*^2^ > 2σ(*F*^2^)] = 0.044	H atoms treated by a mixture of independent and constrained refinement
*wR*(*F*^2^) = 0.125	*w* = 1/[σ^2^(*F*_o_^2^) + (0.0517*P*)^2^ + 0.515*P*] where *P* = (*F*_o_^2^ + 2*F*_c_^2^)/3
*S* = 1.05	(Δ/σ)_max_ < 0.001
4655 reflections	Δρ_max_ = 0.34 e Å^−^^3^
350 parameters	Δρ_min_ = −0.34 e Å^−^^3^
2 restraints	Extinction correction: *SHELXL97* (Sheldrick, 2008), Fc^*^=kFc[1+0.001xFc^2^λ^3^/sin(2θ)]^-1/4^
Primary atom site location: structure-invariant direct methods	Extinction coefficient: 0.0104 (5)

### Special details

**Table:**

Geometry. All esds (except the esd in the dihedral angle between two l.s. planes) are estimated using the full covariance matrix. The cell esds are taken into account individually in the estimation of esds in distances, angles and torsion angles; correlations between esds in cell parameters are only used when they are defined by crystal symmetry. An approximate (isotropic) treatment of cell esds is used for estimating esds involving l.s. planes.
Refinement. Refinement of *F*^2^ against ALL reflections. The weighted *R*-factor wR and goodness of fit *S* are based on *F*^2^, conventional *R*-factors *R* are based on *F*, with *F* set to zero for negative *F*^2^. The threshold expression of *F*^2^ > σ(*F*^2^) is used only for calculating *R*-factors(gt) etc. and is not relevant to the choice of reflections for refinement. *R*-factors based on *F*^2^ are statistically about twice as large as those based on *F*, and *R*- factors based on ALL data will be even larger.

### Fractional atomic coordinates and isotropic or equivalent isotropic displacement parameters (Å^2^)

**Table:**

	*x*	*y*	*z*	*U*_iso_*/*U*_eq_	
Cl1	0.13114 (11)	0.65487 (7)	0.19920 (7)	0.0951 (3)	
S1	0.12434 (7)	0.19435 (5)	−0.03688 (6)	0.0571 (2)	
O1	−0.02885 (19)	0.11806 (14)	−0.03785 (19)	0.0766 (6)	
O2	0.1928 (2)	0.22241 (16)	−0.13596 (16)	0.0727 (5)	
O3	0.45459 (19)	0.26268 (14)	0.02647 (17)	0.0689 (5)	
N1	0.2241 (2)	0.12840 (16)	0.03850 (19)	0.0566 (5)	
H1N	0.172 (3)	0.0617 (17)	0.054 (2)	0.068*	
C1	0.1337 (2)	0.32370 (18)	0.0343 (2)	0.0515 (5)	
C2	0.0252 (3)	0.3229 (2)	0.1058 (2)	0.0633 (7)	
H2	−0.0481	0.2532	0.1196	0.076*	
C3	0.0251 (3)	0.4250 (2)	0.1567 (2)	0.0696 (7)	
H3	−0.0484	0.4255	0.2047	0.084*	
C4	0.1352 (3)	0.5262 (2)	0.1355 (2)	0.0625 (6)	
C5	0.2456 (3)	0.5282 (2)	0.0662 (2)	0.0629 (7)	
H5	0.3200	0.5980	0.0543	0.075*	
C6	0.2461 (3)	0.42605 (19)	0.0140 (2)	0.0574 (6)	
H6	0.3202	0.4259	−0.0337	0.069*	
C7	0.3829 (2)	0.16862 (19)	0.0569 (2)	0.0520 (5)	
C8	0.4541 (2)	0.09067 (19)	0.11647 (19)	0.0491 (5)	
C9	0.6099 (3)	0.1097 (2)	0.1104 (2)	0.0653 (7)	
H9	0.6662	0.1704	0.0708	0.078*	
C10	0.6821 (3)	0.0392 (3)	0.1626 (3)	0.0761 (8)	
H10	0.7867	0.0516	0.1570	0.091*	
C11	0.6022 (3)	−0.0483 (2)	0.2222 (3)	0.0710 (7)	
H11	0.6523	−0.0952	0.2577	0.085*	
C12	0.4478 (3)	−0.0677 (3)	0.2304 (3)	0.0768 (8)	
H12	0.3932	−0.1272	0.2719	0.092*	
C13	0.3737 (3)	0.0014 (2)	0.1767 (2)	0.0667 (7)	
H13	0.2685	−0.0125	0.1813	0.080*	
Cl2	0.09586 (10)	1.12238 (7)	0.31722 (8)	0.0928 (3)	
S2	0.15335 (8)	0.68980 (5)	0.55894 (6)	0.0660 (2)	
O4	0.0012 (2)	0.61359 (16)	0.5643 (2)	0.0871 (7)	
O5	0.2464 (3)	0.72946 (19)	0.65492 (17)	0.0895 (7)	
O6	0.4541 (2)	0.75873 (16)	0.47147 (19)	0.0794 (6)	
N2	0.2341 (3)	0.61526 (18)	0.48162 (19)	0.0623 (5)	
H2N	0.171 (3)	0.5535 (19)	0.456 (2)	0.075*	
C14	0.1436 (3)	0.81131 (19)	0.4870 (2)	0.0544 (6)	
C15	0.0225 (3)	0.7997 (2)	0.4114 (2)	0.0640 (7)	
H15	−0.0496	0.7272	0.3963	0.077*	
C16	0.0090 (3)	0.8952 (2)	0.3589 (2)	0.0675 (7)	
H16	−0.0714	0.8881	0.3073	0.081*	
C17	0.1157 (3)	1.0018 (2)	0.3832 (2)	0.0605 (6)	
C18	0.2376 (3)	1.0149 (2)	0.4571 (2)	0.0645 (7)	
H18	0.3097	1.0876	0.4713	0.077*	
C19	0.2515 (3)	0.9182 (2)	0.5102 (2)	0.0602 (6)	
H19	0.3328	0.9253	0.5610	0.072*	
C20	0.3804 (3)	0.6593 (2)	0.4480 (2)	0.0595 (6)	
C21	0.4389 (3)	0.5793 (2)	0.3827 (2)	0.0550 (6)	
C22	0.3646 (3)	0.4623 (2)	0.3640 (3)	0.0712 (7)	
H22	0.2712	0.4285	0.3932	0.085*	
C23	0.4278 (4)	0.3951 (3)	0.3022 (3)	0.0816 (9)	
H23	0.3760	0.3163	0.2896	0.098*	
C24	0.5657 (3)	0.4427 (3)	0.2590 (3)	0.0744 (8)	
H24	0.6075	0.3966	0.2173	0.089*	
C25	0.6410 (4)	0.5582 (3)	0.2777 (3)	0.0829 (9)	
H25	0.7353	0.5911	0.2493	0.100*	
C26	0.5780 (3)	0.6261 (2)	0.3385 (3)	0.0782 (8)	
H26	0.6299	0.7051	0.3502	0.094*	

### Atomic displacement parameters (Å^2^)

**Table:**

	*U*^11^	*U*^22^	*U*^33^	*U*^12^	*U*^13^	*U*^23^
Cl1	0.1252 (7)	0.0788 (5)	0.0934 (6)	0.0542 (5)	−0.0124 (5)	−0.0192 (4)
S1	0.0512 (3)	0.0391 (3)	0.0765 (4)	0.0097 (2)	−0.0101 (3)	0.0026 (2)
O1	0.0526 (9)	0.0425 (8)	0.1251 (18)	0.0060 (7)	−0.0250 (10)	0.0046 (9)
O2	0.0922 (13)	0.0609 (10)	0.0653 (12)	0.0257 (9)	−0.0039 (10)	−0.0025 (9)
O3	0.0507 (9)	0.0505 (9)	0.0989 (15)	0.0043 (7)	0.0056 (9)	0.0139 (9)
N1	0.0419 (9)	0.0415 (9)	0.0829 (15)	0.0082 (8)	−0.0021 (9)	0.0104 (9)
C1	0.0470 (11)	0.0416 (11)	0.0643 (15)	0.0114 (9)	−0.0012 (10)	0.0077 (10)
C2	0.0514 (13)	0.0562 (14)	0.0809 (19)	0.0114 (10)	0.0121 (12)	0.0204 (12)
C3	0.0690 (16)	0.0765 (17)	0.0723 (18)	0.0323 (14)	0.0171 (14)	0.0148 (14)
C4	0.0729 (16)	0.0553 (13)	0.0631 (16)	0.0277 (12)	−0.0078 (13)	0.0007 (11)
C5	0.0656 (15)	0.0402 (12)	0.0769 (18)	0.0068 (10)	0.0027 (13)	0.0069 (11)
C6	0.0552 (13)	0.0447 (12)	0.0689 (16)	0.0082 (10)	0.0109 (12)	0.0075 (11)
C7	0.0431 (11)	0.0459 (12)	0.0637 (15)	0.0089 (9)	0.0024 (10)	−0.0040 (10)
C8	0.0419 (11)	0.0478 (11)	0.0555 (14)	0.0112 (9)	0.0009 (9)	−0.0070 (9)
C9	0.0425 (12)	0.0564 (14)	0.092 (2)	0.0087 (10)	0.0021 (12)	0.0037 (13)
C10	0.0430 (12)	0.0755 (17)	0.110 (3)	0.0201 (12)	−0.0022 (14)	0.0012 (16)
C11	0.0653 (16)	0.0690 (16)	0.082 (2)	0.0296 (13)	−0.0142 (14)	−0.0009 (14)
C12	0.0683 (17)	0.0810 (18)	0.082 (2)	0.0226 (14)	0.0032 (15)	0.0251 (15)
C13	0.0474 (12)	0.0766 (17)	0.0776 (19)	0.0194 (12)	0.0079 (12)	0.0196 (14)
Cl2	0.1041 (6)	0.0891 (5)	0.1031 (7)	0.0515 (5)	0.0222 (5)	0.0280 (4)
S2	0.0791 (4)	0.0521 (3)	0.0653 (4)	0.0152 (3)	0.0167 (3)	0.0026 (3)
O4	0.0882 (14)	0.0571 (10)	0.1117 (17)	0.0069 (10)	0.0492 (13)	0.0039 (10)
O5	0.1332 (19)	0.0795 (13)	0.0582 (12)	0.0377 (13)	−0.0030 (12)	−0.0003 (10)
O6	0.0592 (10)	0.0560 (10)	0.1145 (17)	0.0062 (8)	0.0011 (11)	−0.0095 (10)
N2	0.0630 (12)	0.0497 (11)	0.0712 (15)	0.0112 (9)	0.0106 (11)	−0.0010 (10)
C14	0.0524 (12)	0.0488 (12)	0.0573 (14)	0.0079 (10)	0.0075 (10)	−0.0066 (10)
C15	0.0537 (13)	0.0574 (14)	0.0719 (18)	0.0057 (11)	−0.0020 (12)	−0.0157 (12)
C16	0.0584 (14)	0.0792 (18)	0.0644 (17)	0.0228 (13)	−0.0065 (12)	−0.0102 (13)
C17	0.0597 (14)	0.0617 (14)	0.0651 (16)	0.0240 (11)	0.0123 (12)	0.0038 (12)
C18	0.0587 (14)	0.0476 (13)	0.0794 (18)	0.0046 (10)	0.0034 (13)	−0.0032 (12)
C19	0.0509 (12)	0.0560 (13)	0.0666 (16)	0.0079 (10)	−0.0058 (11)	−0.0064 (11)
C20	0.0540 (13)	0.0533 (13)	0.0672 (16)	0.0113 (11)	−0.0038 (12)	0.0062 (11)
C21	0.0512 (12)	0.0522 (12)	0.0601 (15)	0.0141 (10)	−0.0022 (11)	0.0103 (10)
C22	0.0571 (14)	0.0602 (15)	0.094 (2)	0.0124 (12)	0.0125 (14)	0.0022 (14)
C23	0.0749 (18)	0.0602 (16)	0.107 (3)	0.0148 (14)	0.0140 (17)	−0.0050 (15)
C24	0.0730 (17)	0.0806 (19)	0.075 (2)	0.0314 (15)	0.0052 (15)	0.0013 (15)
C25	0.0692 (17)	0.082 (2)	0.096 (2)	0.0165 (15)	0.0253 (17)	0.0109 (17)
C26	0.0694 (17)	0.0589 (15)	0.100 (2)	0.0066 (13)	0.0172 (16)	0.0088 (15)

### Geometric parameters (Å, °)

**Table:**

Cl1—C4	1.737 (3)	Cl2—C17	1.734 (3)
S1—O2	1.424 (2)	S2—O5	1.415 (2)
S1—O1	1.4251 (18)	S2—O4	1.425 (2)
S1—N1	1.645 (2)	S2—N2	1.653 (2)
S1—C1	1.753 (2)	S2—C14	1.753 (3)
O3—C7	1.208 (3)	O6—C20	1.204 (3)
N1—C7	1.388 (3)	N2—C20	1.378 (3)
N1—H1N	0.836 (17)	N2—H2N	0.834 (17)
C1—C2	1.374 (4)	C14—C19	1.379 (3)
C1—C6	1.387 (3)	C14—C15	1.383 (4)
C2—C3	1.373 (4)	C15—C16	1.368 (4)
C2—H2	0.9300	C15—H15	0.9300
C3—C4	1.370 (4)	C16—C17	1.373 (4)
C3—H3	0.9300	C16—H16	0.9300
C4—C5	1.365 (4)	C17—C18	1.372 (4)
C5—C6	1.382 (3)	C18—C19	1.386 (4)
C5—H5	0.9300	C18—H18	0.9300
C6—H6	0.9300	C19—H19	0.9300
C7—C8	1.480 (3)	C20—C21	1.486 (4)
C8—C13	1.375 (3)	C21—C22	1.375 (4)
C8—C9	1.380 (3)	C21—C26	1.381 (4)
C9—C10	1.372 (4)	C22—C23	1.376 (4)
C9—H9	0.9300	C22—H22	0.9300
C10—C11	1.355 (4)	C23—C24	1.368 (4)
C10—H10	0.9300	C23—H23	0.9300
C11—C12	1.370 (4)	C24—C25	1.361 (4)
C11—H11	0.9300	C24—H24	0.9300
C12—C13	1.379 (4)	C25—C26	1.375 (4)
C12—H12	0.9300	C25—H25	0.9300
C13—H13	0.9300	C26—H26	0.9300
			
O2—S1—O1	119.10 (13)	O5—S2—O4	119.32 (15)
O2—S1—N1	110.02 (12)	O5—S2—N2	110.30 (14)
O1—S1—N1	103.39 (10)	O4—S2—N2	103.33 (12)
O2—S1—C1	109.16 (11)	O5—S2—C14	108.61 (12)
O1—S1—C1	108.42 (12)	O4—S2—C14	108.37 (13)
N1—S1—C1	105.90 (11)	N2—S2—C14	106.11 (12)
C7—N1—S1	123.68 (17)	C20—N2—S2	123.63 (18)
C7—N1—H1N	122.9 (19)	C20—N2—H2N	123 (2)
S1—N1—H1N	112.3 (19)	S2—N2—H2N	112 (2)
C2—C1—C6	121.1 (2)	C19—C14—C15	120.8 (2)
C2—C1—S1	119.31 (18)	C19—C14—S2	119.9 (2)
C6—C1—S1	119.5 (2)	C15—C14—S2	119.27 (18)
C3—C2—C1	119.9 (2)	C16—C15—C14	119.8 (2)
C3—C2—H2	120.1	C16—C15—H15	120.1
C1—C2—H2	120.1	C14—C15—H15	120.1
C4—C3—C2	118.9 (3)	C15—C16—C17	119.2 (2)
C4—C3—H3	120.6	C15—C16—H16	120.4
C2—C3—H3	120.6	C17—C16—H16	120.4
C5—C4—C3	122.0 (3)	C18—C17—C16	121.9 (2)
C5—C4—Cl1	119.9 (2)	C18—C17—Cl2	119.4 (2)
C3—C4—Cl1	118.1 (2)	C16—C17—Cl2	118.7 (2)
C4—C5—C6	119.6 (2)	C17—C18—C19	118.9 (2)
C4—C5—H5	120.2	C17—C18—H18	120.5
C6—C5—H5	120.2	C19—C18—H18	120.5
C5—C6—C1	118.5 (2)	C14—C19—C18	119.3 (2)
C5—C6—H6	120.7	C14—C19—H19	120.3
C1—C6—H6	120.7	C18—C19—H19	120.3
O3—C7—N1	120.4 (2)	O6—C20—N2	119.9 (3)
O3—C7—C8	123.8 (2)	O6—C20—C21	123.1 (2)
N1—C7—C8	115.85 (19)	N2—C20—C21	117.0 (2)
C13—C8—C9	118.9 (2)	C22—C21—C26	118.1 (3)
C13—C8—C7	123.6 (2)	C22—C21—C20	124.5 (2)
C9—C8—C7	117.5 (2)	C26—C21—C20	117.4 (2)
C10—C9—C8	120.2 (3)	C21—C22—C23	120.3 (3)
C10—C9—H9	119.9	C21—C22—H22	119.8
C8—C9—H9	119.9	C23—C22—H22	119.8
C11—C10—C9	120.6 (2)	C24—C23—C22	120.9 (3)
C11—C10—H10	119.7	C24—C23—H23	119.5
C9—C10—H10	119.7	C22—C23—H23	119.5
C10—C11—C12	120.1 (3)	C25—C24—C23	119.3 (3)
C10—C11—H11	119.9	C25—C24—H24	120.4
C12—C11—H11	119.9	C23—C24—H24	120.4
C11—C12—C13	119.8 (3)	C24—C25—C26	120.1 (3)
C11—C12—H12	120.1	C24—C25—H25	119.9
C13—C12—H12	120.1	C26—C25—H25	119.9
C8—C13—C12	120.4 (2)	C25—C26—C21	121.2 (3)
C8—C13—H13	119.8	C25—C26—H26	119.4
C12—C13—H13	119.8	C21—C26—H26	119.4
			
O2—S1—N1—C7	47.9 (2)	O5—S2—N2—C20	−56.1 (3)
O1—S1—N1—C7	176.1 (2)	O4—S2—N2—C20	175.3 (2)
C1—S1—N1—C7	−70.0 (2)	C14—S2—N2—C20	61.3 (2)
O2—S1—C1—C2	152.8 (2)	O5—S2—C14—C19	18.5 (3)
O1—S1—C1—C2	21.6 (2)	O4—S2—C14—C19	149.5 (2)
N1—S1—C1—C2	−88.8 (2)	N2—S2—C14—C19	−100.1 (2)
O2—S1—C1—C6	−24.3 (2)	O5—S2—C14—C15	−158.2 (2)
O1—S1—C1—C6	−155.5 (2)	O4—S2—C14—C15	−27.2 (2)
N1—S1—C1—C6	94.1 (2)	N2—S2—C14—C15	83.3 (2)
C6—C1—C2—C3	1.4 (4)	C19—C14—C15—C16	−0.1 (4)
S1—C1—C2—C3	−175.7 (2)	S2—C14—C15—C16	176.5 (2)
C1—C2—C3—C4	−0.6 (4)	C14—C15—C16—C17	−0.7 (4)
C2—C3—C4—C5	−0.6 (4)	C15—C16—C17—C18	1.5 (4)
C2—C3—C4—Cl1	179.0 (2)	C15—C16—C17—Cl2	−179.2 (2)
C3—C4—C5—C6	1.1 (4)	C16—C17—C18—C19	−1.3 (4)
Cl1—C4—C5—C6	−178.5 (2)	Cl2—C17—C18—C19	179.3 (2)
C4—C5—C6—C1	−0.3 (4)	C15—C14—C19—C18	0.2 (4)
C2—C1—C6—C5	−0.9 (4)	S2—C14—C19—C18	−176.4 (2)
S1—C1—C6—C5	176.14 (19)	C17—C18—C19—C14	0.5 (4)
S1—N1—C7—O3	7.9 (4)	S2—N2—C20—O6	−3.1 (4)
S1—N1—C7—C8	−173.19 (17)	S2—N2—C20—C21	177.37 (18)
O3—C7—C8—C13	160.9 (3)	O6—C20—C21—C22	172.9 (3)
N1—C7—C8—C13	−18.0 (4)	N2—C20—C21—C22	−7.6 (4)
O3—C7—C8—C9	−19.1 (4)	O6—C20—C21—C26	−6.7 (4)
N1—C7—C8—C9	162.0 (2)	N2—C20—C21—C26	172.9 (2)
C13—C8—C9—C10	0.9 (4)	C26—C21—C22—C23	−0.5 (4)
C7—C8—C9—C10	−179.0 (3)	C20—C21—C22—C23	179.9 (3)
C8—C9—C10—C11	−1.3 (5)	C21—C22—C23—C24	0.6 (5)
C9—C10—C11—C12	0.5 (5)	C22—C23—C24—C25	0.0 (5)
C10—C11—C12—C13	0.7 (5)	C23—C24—C25—C26	−0.6 (5)
C9—C8—C13—C12	0.2 (4)	C24—C25—C26—C21	0.7 (5)
C7—C8—C13—C12	−179.8 (3)	C22—C21—C26—C25	−0.1 (5)
C11—C12—C13—C8	−1.0 (5)	C20—C21—C26—C25	179.5 (3)

### Hydrogen-bond geometry (Å, °)

**Table:**

*D*—H···*A*	*D*—H	H···*A*	*D*···*A*	*D*—H···*A*
N1—H1N···O1^i^	0.84 (2)	2.16 (2)	2.967 (3)	161 (3)
N2—H2N···O4^ii^	0.83 (2)	2.15 (2)	2.962 (3)	164 (3)

Symmetry codes: (i) −*x*, −*y*, −*z*; (ii) −*x*, −*y*+1, −*z*+1.
